# Assessing Fast Structure Formation Processes in Isotactic Polypropylene with a Combination of Nanofocus X-ray Diffraction and In Situ Nanocalorimetry

**DOI:** 10.3390/nano11102652

**Published:** 2021-10-09

**Authors:** Yaroslav Odarchenko, Martin Rosenthal, Jaime J. Hernandez, David Doblas, Emanuela Di Cola, Mikhail Soloviev, Dimitri A. Ivanov

**Affiliations:** 1 Institut de Sciences des Matériaux de Mulhouse-IS2M, CNRS UMR 7361, Jean Starcky, 15, F-68057 Mulhouse, France; yaro@finden.co.uk (Y.O.); jaime.hernandez@imdea.org (J.J.H.); david.doblas-jimenez@xfel.eu (D.D.); 2 Department of Biological Sciences, Royal Holloway University of London, Egham, Surrey TW20 0EX, UK; 3 European Synchrotron Radiation Facility (ESRF), 38043 Grenoble, France; dicola@sas-analysis.eu; 4 Faculty of Chemistry, Lomonosov Moscow State University (MSU), 1 Leninskie Gory, 119991 Moscow, Russia; 5 Institute of Problems of Chemical Physics, Russian Academy of Sciences, 142432 Chernogolovka, Russia

**Keywords:** nanocalorimetry, nanofocus X-ray diffraction, isotactic polypropylene, mesophase, crystallization

## Abstract

A combination of in situ nanocalorimetry with simultaneous nanofocus 2D Wide-Angle X-ray Scattering (WAXS) was used to study polymorphic behaviour and structure formation in a single micro-drop of isotactic polypropylene (iPP) with defined thermal history. We were able to generate, detect, and characterize a number of different iPP morphologies using our custom-built ultrafast chip-based nanocalorimetry instrument designed for use with the European Synchrotron Radiation Facility (ESRF) high intensity nanofocus X-ray beamline facility. The detected iPP morphologies included monoclinic alpha-phase crystals, mesophase, and mixed morphologies with different mesophase/crystalline compositional ratios. Monoclinic crystals formed from the mesophase became unstable at heating rates above 40 K s^−1^ and showed melting temperatures as low as ~30 K below those measured for iPP crystals formed by slow cooling. We also studied the real-time melt crystallization of nanogram-sized iPP samples. Our analysis revealed a mesophase nucleation time of around 1 s and the co-existence of mesophase and growing disordered crystals at high supercooling ≤328 K. The further increase of the iPP crystallization temperature to 338 K changed nucleation from homogeneous to heterogeneous. No mesophase was detected above 348 K. Low supercooling (≥378 K) led to the continuous growth of the alpha-phase crystals. In conclusion, we have, for the first time, measured the mesophase nucleation time of supercooled iPP melted under isothermal crystallization conditions using a dedicated experimental setup designed to allow simultaneous ultrafast chip-based nanocalorimetry and nanofocus X-ray diffraction analyses. We also provided experimental evidence that upon heating, the mesophase converts directly into thermodynamically stable monoclinic alpha-phase crystals via perfection and reorganization and not via partial melting. The complex phase behaviour of iPP and its dependence on both crystallization temperature and time is presented here using a time–temperature–transformation (TTT) diagram.

## 1. Introduction

Isotactic polypropylene (iPP) combines simple chemical architecture with distinctive polymorphic behaviour and is one of the most intensely studied semicrystalline polymers. When iPP is cooled down slowly from the melt it can often yield several crystalline polymorphs such as α-monoclinic, β-hexagonal, and γ-triclinic. Quenching iPP melt (cooling rates higher than approximately 100 K s^−1^) may also produce a mesophase consisting of conformationally disordered crystals [1,2]. The mesophase stability and its transition to the thermodynamically stable alpha-phase has been widely investigated in the past [3,4,5]. The results of temperature-resolved X-ray scattering experiments that have been reported previously for slowly heated iPP (rates below 0.1 K s^−1^) or iPP annealed at elevated temperatures yielded mesophase transition temperatures of between 350 K and 360 K [6,7]. However, some important aspects of the temperature dependence of the reorganization processes in mesophase that happen upon heating are not fully understood. For example, whether the mesophase converts into crystals directly or via partial melting and/or reorganization remains unknown.

Nanocalorimetry allows the study of minute quantities of material at very high heating/cooling rates up to approximately 10^6^ K s^−1^ [8], thus providing an advantage over conventional differential scanning calorimetry (DSC). Traditional commercially available DSC instruments are typically capable of heating/cooling rates of below approximately 10 K s^−1^ and are not suitable for studying fast reorganization processes. Following the earlier developments by L. Allen et al. [9], chip-based calorimetry emerged as a powerful technique for the analysis of different types of materials such as metals [8,10,11], explosives [12], or polymers [13,14]. Mileva et al. used chip-based calorimetry analysis to study the annealing of fully amorphous glassy iPP at 300 K and reported a mesophase formation time of around 0.1 s and determined its melting temperature for the first time [3,4]. However, because of the complex phase behaviour and intrinsic polymorphism of iPP, calorimetry studies alone often yield insufficient data. Therefore, additional structural information, such as from wide angle X-ray Scattering (WAXS), Infrared (IR) spectroscopy, or solid-state nuclear magnetic resonance (NMR) analyses, is often required to interpret the results of calorimetry studies.

Combining fast calorimetry with other X-ray diffraction characterization techniques remains a challenge, although some successful experiments employing Fourier transform infrared (FTIR) spectroscopy for studying natural polymers or chromatography for studying propylene copolymers have been reported in the past [15,16]. The first successful combination of in situ nanocalorimetry with X-ray diffraction (XRD) was by Vlassak et al. for studying effects of the composition and quenching rate on Au-Cu-Si metallic glasses [17]. Advances in synchrotron optics enabled focusing an X-ray beam to micro- and nanosize, which is beneficial for the study of small samples, which are often required for chip-based nanocalorimetry analysis. A high intensity nanofocused X-ray beamline facility available at the ESRF, which has a sub-200 nm beam and working distances of up to 4 mm [18], has allowed us to characterize morphologies formed by several flexible and semi-rigid chain polymers in the past [19,20,21,22]. Subsequently a nanocalorimeter was built and integrated in the nanodiffraction setup of the ID13 beamline at ESRF [23,24]. This combination allowed us to not only control and change temperature but also to directly correlate temperature changes with spatially resolved X-ray absorption measurements for the first time. Our in situ nanocalorimetry stage was originally tested by imaging a nanogram size ultra-pure metal particle [23]. We also showed that the dynamic thermal response of the sample measured under quasi-isothermal conditions during X-ray beam exposures provides additional information such as the specific fusion heat for the phase transitions occurring in the sample. We have since conducted simultaneous calorimetric and X-ray scattering analyses of self-assembled carbohydrate-functionalized gold nanoparticles [25], high-energy materials such as CL-20/HMX and CL-20/TNT [26], and a semirigid-chain polymer poly(trimethylene terephthalate) [27,28]. As far as we are aware, the only other study where a combination of in situ nanocalorimetry and X-ray diffraction has been applied to study polymers was a study determining the isothermal crystallization of polyethylene and polyamide that yielded a fast mesomorphic phase formation time of less than a second for polyamide 11 [29]. Previous research on iPP was largely limited to ex situ measurements of quenched drops of iPP using a commercially available fast scanning chip calorimeter and a microfocus WAXS setup [30]. There, cooling rates of 40 K s^−1^ and 200 K s^−1^ were used, which resulted in the formation of monoclinic crystals or a mesophase, respectively.

The polymorphic behaviour of materials can be conveniently visualised using continuous cooling transformation (CCT), continuous heating transformation (CHT) and time-temperature-transformation (TTT) diagrams [31]. Although, the latter approach is widely utilised in studies of metals, it has rarely been applied to polymers. To the best of our knowledge, only a few CCT studies on iPP and its copolymers have been reported to date [2,32,33,34]. For example, Cavallo et al. showed that the mesomorphic structure prevails above the critical cooling rate, which decreases with increasing ethylene content from approximately 200 K s^−1^ for a homopolymer to less than 70 K s^−1^ for a copolymer with 7.3 mol % of ethylene [2]. Perez and co-workers showed that the rate of mesophase formation can be tuned, covering four orders of magnitude by randomly adding 1-pentene or 1-octene units up to 8.9 mol % in iPP [33,34]. Choi et al. found that applied spinline stress can enhance alpha-phase formation while preventing mesophase formation under severe quenching conditions (up to 10^4^ K s^−1^) [32]. TTT diagrams are useful for understanding the kinetics of phase transitions in isothermally cooled materials. Typically, such data can be generated by performing isothermal crystallization studies from polymer melts. De Santis et al. conducted an isothermal crystallization of iPP, measured the time required to reach the onset of the exothermic peak at a range of different temperatures, and concluded that the solidification mechanism for iPP changes at around 45 °C [35]. Later, such bimodal crystallization behaviour was confirmed by Silvestre et al. [36] using a combination of calorimetric analysis with the ex situ WAXS of thin f iPP films that had been rapidly quenched from the melt into water. The authors argued that the crystallization of the iPP mesophase by homogeneous nucleation occurs at temperatures between Tg and 333 K, whilst higher temperatures promote a heterogeneous nucleation of the monoclinic crystals. However, no data on the polymer phase composition or their dependence on the crystallization time were reported for supercooled iPP melts [36]. In this work, we set out to conduct a comprehensive in situ structural analysis of iPP polymorphism using our chip-based nanocalorimeter instrument combined with nanofocus 2D WAXS. To this end, from their characteristic X-ray diffraction patterns, we have identified and quantified the formation of crystalline, mesomorphic, and amorphous phases in iPP as a function of the isothermal solidification temperature, time, and scanning rates of up to 3000 K s^−1^.

## 2. Materials and Methods

**Figure 1:**
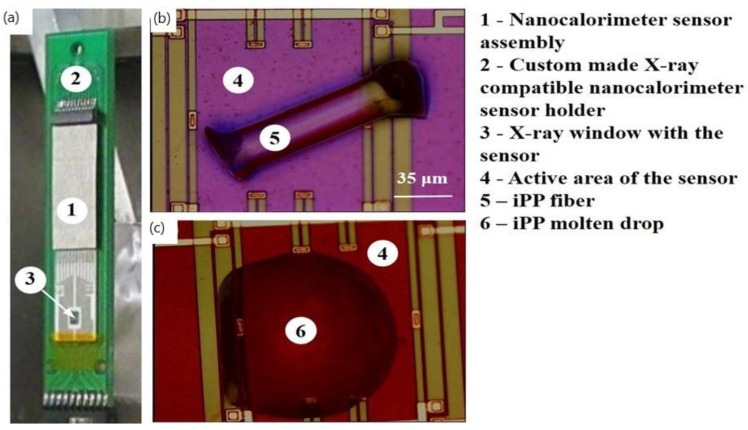
Nanocalorimeter sensor assembly used for nanofocus 2D Wide-Angle X-ray Scattering (WAXS). (**a**) Sensor; (**b**) a fragment of isotactic polypropylene (iPP) fibre deposited on the active area of the sensor; (**c**) a drop of molten iPP formed after the first heating cycle.

The melt-spun iPP filaments with well-defined diameters and crystallinity with Mn = 58 kg mol^−1^ and Mw = 161 kg mol^−1^ were provided by Total Petrochemicals. A detailed characterisation, including the degree of crystallinity of the iPP samples used in this work, has been reported elsewhere [37]. The known thickness of the polymer fibres allowed us to estimate the sample weight from their cylindrical geometry and iPP density of 0.90 g/cm^−3^. The WAXS patterns indicate the mesomorphic state of iPP samples (Figure S1). A custom-built ultrafast microelectromechanical systems (MEMS)-based nanocalorimeter instrument (Figure 1a) designed for use with the ESRF high intensity nanofocus X-ray beamline facility was used to generate and study iPP morphologies with a defined thermal history. Small fragments of melt-spun iPP fibres (Figure 1b) typically weighing between 30 ng and 40 ng were positioned within the active area of the calorimetric sensor using a micromanipulator. iPP samples were melted and were then crystallized under defined cooling conditions to produce uniform highly crystalline iPP drops (Figure 1c). The crystallization rates that were used are specified in the temperature–time (TT) profiles. The nanobeam X-ray diffraction measurements were conducted at the ID13 beamline of the ESRF (Grenoble, France). X-ray photons with an energy of 14.92 keV were used. The footprint of the beam on a sample of about 150 × 150 nm^2^ was achieved using silicon-based compound refractive nano-focusing X-ray lenses with a focal length of 14 mm [18]. Two-dimensional diffraction patterns were collected with a 2D Frelon camera with 50 × 50 µm pixels. Data acquisition was conducted using 2 × 2 or 4 × 4 binning. The norm of the scattering vector **s**:

|**s**| = 2sin(θ)/λ (1)

was calibrated using several corundum reflections. The calorimetric measurements were performed with MEMS-type sensors XEN-39392 (Xensor Integration B.V.EJ Delfgauw, The Netherlands) assembled on a ceramic flat plate. The active area of the sensor Si_x_N_y_ membrane was 100 × 100 µm^2^.

The thickness of the silicon nitride membrane of the integrated sensor was 1 µm, and it was transparent to the X-ray beam. For 2D WAXS analysis of the isothermal melt crystallization experiments an acquisition time of 1 s was used. The binning of 4 × 4 pixels was selected to achieve a compromise between a high time resolution and the collected number of counts. The detector saving time of 0.4 s resulted in a WAXS sampling rate of 1.4 s.

## 3. Results

**Figure 2:**
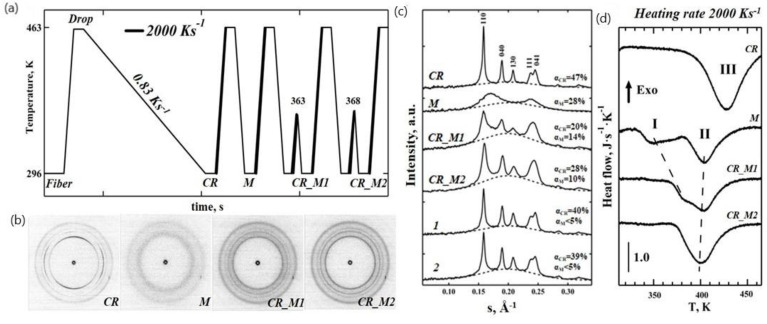
Mesophase–crystal phase transition in iPP. (**a**) Temperature–time (TT) profiles used to obtain different iPP morphologies; (**b**) two-dimensional WAXS patterns corresponding to the crystalline (*CR*), mesomorphic (*M*), and crystalline–mesomorphic (*CRM1, CRM2*) states; (**c**) The 1D WAXS profiles of iPP (Y axis—intensity, a.u., X axis—the modulus of the scattering vector, Å^−1^). The polymer morphologies are indicated above each of the convoluted profiles and include crystalline (*CR*), mesomorphic (*M*), and crystalline–mesomorphic (*CR_M1, CR_M2, 1, 2*) morphologies. Crystalline and mesophase fractions (α_CR_, α_M_) are specified for each of the morphologies. Dashed lines indicate a contribution of the amorphous fraction. Samples 1 and 2, shown here for comparison, were generated by heating the mesophase to 413 K using heating rates of 0.5 K s^−1^ and 2 K s^−1^ and then quenching the polymer to room temperature. (**d**) Heating endotherms with a constant rate of 2000 K s^−1^ probing the resulting iPP morphologies. All of the endotherms are normalized by the sample mass and heating rate.

The flat sensor was used in transmission mode at the ID13 nanofocus beamline of ESRF (Figure 1a). The iPP samples (Figure 1b) were melted and were then slowly crystallized at a rate of 0.83 K s^−1^ to produce uniform highly crystalline iPP drops (Figure 1c). The TT profiles used to generate various iPP morphologies, including the highly crystalline (CR) morphologies obtained upon the slow cooling from melt and that were used as a reference, the mesomorphic (M) and the mixed crystalline/mesomorphic (CR_M1, CR_M2) morphologies obtained upon the heating of the mesophase, are illustrated in Figure 2a. After preparation, each morphology was analysed using real time calorimetry and nanofocus X-ray diffraction measurements. The 2D WAXS diffractogram corresponding to the CR state is shown in Figure 2b. The reduced 1D WAXS profile of CR exhibits several sharp diffraction peaks that were assigned to the monoclinic phase of iPP (Figure 2c). The nanocalorimetry scan of the CR morphology using a heating rate of 2000 K s^−1^ shows a broad melting peak with a maximum at 427 K (Peak III in Figure 2d). We estimated the degree of crystallinity to be 47% from the decomposed WAXS data. The mesomorphic state of iPP (marked “M” in Figure 2a) was generated by quenching the sample in the molten state from 463 K to 296 K at 2000 K s^−1^. The corresponding 2D WAXS pattern and integrated 1D curve exhibit two broad scattering peaks characteristic for the iPP mesophase and no further crystallographic reflections indicating the absence of a crystalline register (Figure 2b,c). The degree of mesomorphicity in the M state was 28%. The nanocalorimetry analysis of the mesomorphic state reveals two endothermic peaks (I and II in Figure 2d), and their nature has been deduced from the WAXS data.

To determine whether the mesophase converts into monoclinic crystals directly or via partial melting (Peak I in Figure 2d) the mesomorphic polymer in state M was annealed at temperatures of 363 K or 368 K, which are higher than 350 K (the temperature of the maximum of Peak I), then quenched at the rate of 2000 K s^−1^ and analysed by X-ray (Figure 2). The recorded 2D WAXS patterns of the generated crystalline–mesomorphic states CR_M1 and CR_M2 (annealing temperatures 363 K and 368 K, respectively) revealed four diffused isotropic peaks (Figure 2c). These morphologies exhibit much broader peaks compared to the CR state in the 1D WAXS profiles (Figure 2c), as estimated from the full width at half maximum (FWHM) values for the 040 peak that increase from 0.0059 for CR to 0.0107 for CR_M2 and 0.0124 for CR_M1, indicating the presence of disordered nascent crystals. There is also a noticeable shoulder between the peaks at 110 and 040 of the alpha-phase in CR_M1 and CR_M2, with the s value representing the first reflection of the mesophase (see for example Figure S2). These peaks indicate that the CR_M1 and CR_M2 morphologies comprise three different phases: crystalline, mesomorphic, and amorphous. The relative contents of the crystalline and mesophase fractions depended on the temperature. Increasing the temperature from 363 K to 368 K resulted in an increase of the crystalline fraction from 20% to 28% and a reduction of mesophase fraction from 14% to 10%. Therefore, it is reasonable to conclude that the mesophase converts directly into the monoclinic crystal phase via the perfection and reorganisation mechanism, which could be similar to mesophase annealing that has been reported previously [4]. This is supported by the nanocalorimetry data (trace CR_M1 in Figure 2d), where Peak I gradually shifts to a higher temperature. The onset of melting in CR_M1 occurs at lower temperatures compared to the CR state (temperature difference of 30 K) indicating differences in the stability of the alpha-phase crystals. The crystalline content in the CR_M1 and CR_M2 states as detected by WAXS analysis is reduced to 20% and 28%, respectively, compared to 47% in the CR state (Figure 2c). These results prove the existence of thermodynamically less stable disordered crystals in the CR_M1 and CR_M2 states compared to in the CR form.

**Figure 3:**
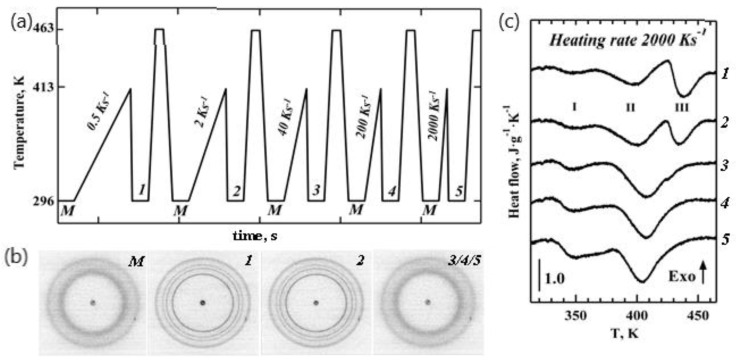
Effect of the heating rate on iPP crystal formation from mesophase. (**a**) TT profile used for the in situ X-ray structural studies; (**b**) two-dimensional WAXS patterns *M 1*–*5* of iPP before and after applying different heating rates. (**c**) Heating endotherm probing states *1*–*5* at a constant rate of 2000 K s^−1^. All of the endotherms are normalized by the sample mass and the heating rates. For the sake of clarity, images *3*, *4,* and *5* of mesomorphic iPP are combined.

To investigate stability of the iPP crystals formed from the mesophase, we subjected our samples to a series of heating cycles with different heating rates. The TT profile that was used is shown in Figure 3a. Diffraction patterns 1–5 (Figure 3b) were generated by heating the mesophase (pattern M) to 413 K, where the heating rates varied from 0.5 K s^−1^ to 2000 K s^−1^, and by then subsequently quenching the polymer to room temperature, marking the initial condition prior to the fast heating experiment.

Several sharp isotropic peaks seen in patterns 1 and 2 (Figure 3b) correspond to the monoclinic phase of iPP, as seen from the 1D WAXS patterns illustrated in Figure 2c (two bottom profiles). The appearance of crystalline morphologies (points 1 and 2 on the TT profile in Figure 3a) is also evident from the presence of additional high temperature Peak III in the corresponding heating endotherms shown in Figure 3c. Peak I in Figure 3c corresponds to mesophase–crystal transition, as concluded above. Therefore, Peaks II and III represent two different populations of alpha-phase iPP crystals obtained upon heating and cooling, respectively, and not the melting of the mesophase. Crystalline morphologies (points 1 and 2 on the TT profile in Figure 3a) show an approximately 7% lower degree of crystallinity compared to the highly crystalline CR state (Figure 2d). Morphologies (points 3, 4, and 5 on the TT profile in Figure 3a) yielded diffraction patterns that were identical to that of the initial mesomorphic state M. Nanocalorimetry traces 3 to 5 in Figure 3c only have Peaks I and II and are similar to the heating endotherms observed for the mesomorphic state M of iPP (Figure 2d).

**Figure 4:**
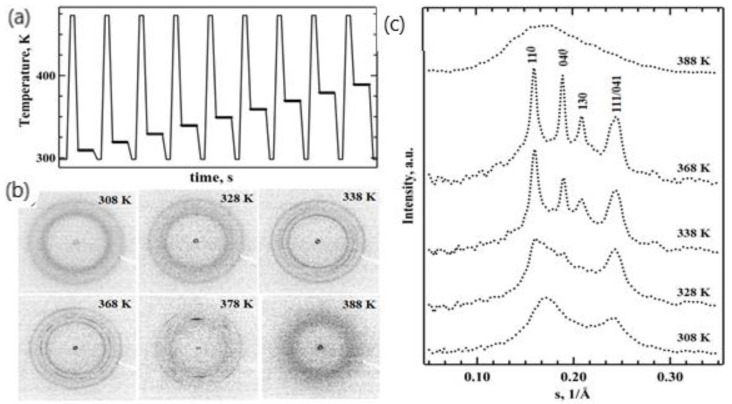
Isothermal melt crystallization of iPP. (**a**) TT profile used for the real-time X-ray structural studies of the isothermal melt crystallization of iPP with a cooling rate of 3000 K s^−1^; (**b**) selected 2D WAXS patterns probing the structure after 7 s at different crystallization temperatures from 308 K to 388 K. (**c**) Reduced 1D WAXS profiles of the resulting iPP morphology.

**Figure 5:**
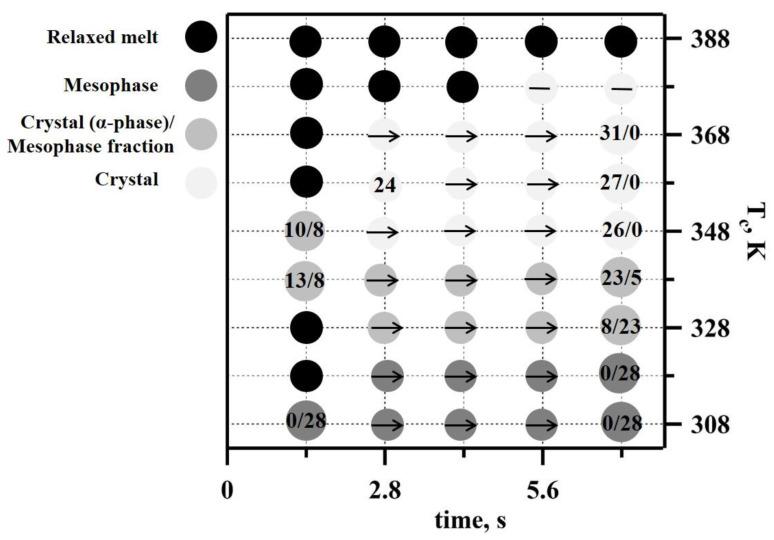
Time–Temperature–Transformation (TTT) diagram for iPP obtained using combined in situ nanocalorimetry and nanfocused X-ray diffraction measurements. Melt, mesophase, and crystalline phases are indicated using different shades of grey. Numbers indicate the calculated crystalline to mesophase fractions. The arrow indicates the same value as the one it is pointing towards.

Another TT profile was used to examine the real-time isothermal melt crystallization of iPP (Figure 4a). A nanogram-sized relaxed melt was quenched at a rate of 3000 K s^−1^ to different annealing temperatures. The annealing process was then monitored for 7 s using nanofocus X-ray diffraction. The temperature profile incorporated multiple crystallization stages (Figure 4a). The first crystallization step was conducted at 308 K, and in each subsequent crystallization step, the temperature was increased by 10 K. The last crystallization step was conducted at 388 K. Selected 2D WAXS patterns and the corresponding 1D-reduced profiles probing iPP morphology are presented in Figure 4. We detected only two diffused peaks at 308 K and 318 K; these indicate a mesophase formation in the iPP samples (exemplified in Figure 4b for 308 K crystallization cycle). The mesophase fraction in iPP crystallized at 308 K was 28% based on the decomposed 1D WAXS profile (from Figure 4c), and the same mesophase content was detected at 318 K. The effect of time and temperature on structure formation is summarised in a representative TTT diagram (Figure 5). The increase in the crystallization temperature from 308 K to 318 K delayed the mesophase formation by approximately one second (Figure 5).

The time-scale of the polymer isothermal structure formation did not change with the further increase of the crystallization temperature to 328 K. However, the 1D WAXS profiles recorded at 328 K and above (Figure 4a) indicate improved iPP ordering compared to those recorded at 308 K and 318 K (Figure 4c). A separate reflection around s = 0.02 Å^−1^ that can be indexed as a 040 peak becomes distinguishable in the iPP crystallized at 328 K (Figure 4b), indicating the co-existence of highly disordered growing crystals and the mesophase (Figure 4c). Additionally, the first broad peak of the mesophase shifted toward smaller angles and narrowed due to the presence of disordered crystals. Importantly, the mesophase fraction decreased by approximately 5% in comparison to the samples crystallized at 318 K (Figure 5). Crystallization conducted at 338 K yielded prominent crystalline peaks in the 1D WAXS profiles (Figure 4c). Such patterns are typical for alpha-phase crystals of isotactic polypropylene, and therefore, the peaks were indexed using unit cells with monoclinic symmetry. The crystalline fraction increased with the crystallization time from 13 to 23%, while the mesophase fraction decreased from 8 to 5% (Figure 5). A further increase of the crystallization temperature to 348 K only affected the alpha-phase crystals fraction compared to 338 K at 1.4 s. The degree of crystallinity of the generated iPP morphology measured after 7 s reached 26%, whilst no mesophase was present. At 358 and 368 K, the formation of early stage crystals was further delayed by one second, and no mesophase was observed (Figure 5). The degree of crystallinity increased from 24% to 27% in the first 2.8 s at 358 K. At 368 K, a crystallinity value of 31% was detected at 1.4 s, and it remained stable during the dwelling time. The change in the ratio of the peaks at 040 and 010 indicates further improvement in the molecular packing and therefore the thermodynamic stability of monoclinic crystals (Figure 4c). The appearance of supercooled early stage crystals below 150 nm in size was delayed by more than 5 s at 378 K (Figure 5) and was followed by the appearance of highly oriented crystalline reflections (Figure 4b) that are pertinent to the formation of the spherulitic morphology, similar to those observed for slowly cooled iPP, i.e., the CR in Figure 2b. Finally, crystallization at 388 K extended the crystal formation time beyond the 7 s long recording. From the literature, it is well known that the delay in crystallization during heterogeneous crystal formation observed at low supercooling is due to the slow crystallization rate and not due to the nuclei formation that is characteristic of a homogeneous nucleation process [35,36,38]. The slow crystallization time (>7 s) observed in this work for 388 K is in agreement with the recent study of iPP isothermal crystallization performed using fast scanning calorimetry [38], where the reported trend of crystallization time over the range of temperatures that were measured (273 K to 383 K) suggested a crystallization time of approximately 5 s if the temperature was increased to 388 K. As a result, only the isotropic broad diffused halo corresponding to the early stage iPP crystals below 150 nm in size was recorded (Figure 4c). Different spots on the samples were used to minimise beam-induced damage to the sample within the acquisition time of the experiment.

## 4. Discussion

The nanocalorimeter instrument used in this study can efficiently perform the thermal analysis of a very small quantity of material. The greatly increased sensitivity of the instrument is because much higher heating rates are achievable compared to conventional DSC. The nanocalorimetry sensor consists of a suspended micrometer-thick silicon-rich Si_x_N_y_ membrane that contains integrated resistive heating elements and thermocouple hot junctions located within 50–100 µm of the heater strips (Figure 1). The lightweight sensor design provides low thermal inertia that, in combination with the small time constant of the sensor, enables extremely fast heating rates of between 10^2^ and 10^6^ K s^−1^. We have previously shown the feasibility of the combined in situ nanocalorimetry with nanofocus X-ray measurements using an indium particle, where we applied both the DC and AC modes [23]. In this work, the DC mode was used to control fast structure formation in isotactic polypropylene. The time resolution of the 2D WAXS detector is this study was contingent upon the acquisition time required to obtain enough counts for a micro-drop of polyolefin polymer with considerably smaller X-ray scattering power, unlike, e.g., indium [23].

The use of a nanosized beam provides a unique advantage over conventional WAXS by allowing the study of local crystal organisation with high spatial resolution. Using nanofocus 2D WAXS, we were able, for example, to determine that the poly(propylene adipate) crystals form spirals and not helicoids, giving rise to unique non-radial growth [39]. In this work, the nanofocus WAXS patterns revealed diffraction peaks from oriented crystals (CR in Figure 2b) because the beam size (150 nm) was comparable with the dimensions of lamellae crystals in a spherulitic superstructure. The formation of alpha-phase crystals following the slow cooling regime of 0.83 K s^−1^ (Figure 2), similar to the regime used in conventional DSC, is in agreement with the results that we reported previously for polymer crystallized in bulk at a rate of 0.05 K s^−1^ [40]. The broad endothermic peak with onset at 397 K and a maximum peak at 427 K corresponds to the melting temperature of the alpha-phase crystals and matches the temperature determined previously with help of the DSC [37]. The crystallinity degree of 47% from WAXS analysis is in good agreement with the value of 41% obtained from the melting endotherm assuming an enthalpy change of 165 J g^−1^ for a 100% crystalline sample [41]. The presence of two endothermic peaks observed by nanocalorimetry (marked I and II in Figure 2d) is similar to that reported previously for the mesomorphic iPP prepared from the fully amorphous material at 300 K for 10 s–60 s and analysed with heating rates ranging between 500 K s^−1^ and 10^4^ K s^−1^ [3,4]. Because of the absence of clear exothermic peaks in those studies, Peaks I and II were believed to be related to the mesophases of different compositions [4], and the onset temperature of Peak II, which was higher than ~360 K, was previously attributed to the superheating of the mesomorphic state [4]. Unlike past studies, here, we were able to experimentally confirm the nature of Peak I (mesophase-crystal phase transition) and Peak II (melting of the crystalline phase) from the simultaneous 2D WAXS analyses, and this has verified our interpretation of the heating endotherms generated with nanocalorimetry. Our observation that the mesophase converts directly into the monoclinic crystals in iPP is also in agreement with the recent work by Li et al., where a similar phenomenon was observed using a combination of IR and X-ray scattering [42]. The temperature of 413 K was chosen for the TT profile in Figure 3a and was based on the maximum amount of heat flux from Peak II, corresponding to melting of the iPP crystals formed from the mesophase upon heating (trace M in Figure 2d). The detected value of ~437 K for Peak III is in good agreement with the melting temperature of the CR state (Figure 2d). Peak III is absent in traces 3–5 because heating at rates above 40 K s^−1^ do not provide sufficient time for crystals to form and/or to grow. This explains the noticeable decrease of ~30 K in the melting temperature of the disordered crystals formed from the mesophase in comparison to those measured for the iPP crystals formed by slow cooling (Peak II in Figure S3).

In the past, comparable times of 0.1 s to 0.3 s for mesophase formation during the isothermal crystallization of iPP were deduced from the position of the exothermic peaks recorded with fast differential scanning calorimetry [36]. However, at high cooling rates such as 2000 K s^−1^ [36], thermoanalytical curves (the crystallization endotherms) do not allow for differentiation between the mesophase and mixed mesophase-crystalline morphologies [36,38] that could otherwise be detected by X-ray diffraction analysis [1,43]. The subsequent technology developments in thermal analysis have furthered our understanding of mesophase formation in semi-crystalline polymers, but to the best of our knowledge, the work of Nishida et al. remains the only study where time-resolved WAXS analysis of the mesophase formation in iPP has been employed [44]. The authors used a temperature jump hot stage compatible with X-rays and optical microscopy and reported a mesophase delay time of 1.6 s at 293 K [44]. However, the estimated cooling rates were nonlinear and remained limited to under 100 K s^−1^. Furthermore, the slowing rate of cooling at lower temperatures and the non-isothermal conditions could have affected the measurements of the mesophase formation kinetics [44].

In this work, we consistently detected a delay in the mesophase formation of approximately one second under isothermal conditions at a range of supercooling temperatures (Figure 5). We were also able to identify and quantify the mesophase and alpha-phase crystal fractions present in the mixed mesophase-crystalline morphologies (Figure 5). In the past, a similar time scale for mesophase development in iPP was observed in non-isothermal crystallization experiments using a WAXS of polymer film wrapped in aluminium foil [1]. The co-existence of highly disordered supercooled crystals and the mesophase in iPP crystallized at 328 K discovered in this work from WAXS analyses corroborate data on the bimodal crystallization kinetics around 328 K that were obtained using calorimetry alone [35,36]. The nanofocus beam also allowed us to obtain the first independent proof of change in crystal nucleation kinetics from homogeneous to heterogeneous at temperatures above 338 K. Our results support previous studies of supercooled iPP melts, where a similar conclusion was made based solely on the calorimetry data for the temperature of 333 K since polymer composition was only probed using ex situ WAXS [36].

## 5. Conclusions

In conclusion, we reported a successful experiment that was conducted using our dedicated custom-built ultrafast chip-based nanocalorimetry instrument designed for use with the ESRF high intensity nanofocus X-ray beamline. Our experimental approach revealed novel insights into fast structure formation processes in an industrially important semicrystalline polymer. In this work, we reported on the nucleation time and transformation kinetics of the mesophase in iPP and documented its direct transition through perfection and reorganisation into monoclinic crystals upon heating or isothermally and quantified the mesophase to alpha-phase crystal fraction in mixed mesophase-crystalline morphologies of iPP. We showed that disordered iPP crystals formed from the mesophase become unstable at heating rates above 40 K s^−1^ and exhibit melting temperatures ~30 K lower than those measured for crystals formed by slow cooling. The time–temperature–transformation diagram was generated for a wide range of temperatures, from 308 K to 388 K, by performing isothermal melt-solidification of polymer microdrops. The TTT chart revealed the complex phase behaviour of iPP as a function of the crystallization temperature and time. For the first time, we have characterised mesophase nucleation time under isothermal conditions and quantified the mesophase to alpha-phase crystal fraction in mixed mesophase-crystalline morphologies of iPP using a dedicated experimental setup designed to allow simultaneous ultrafast chip-based nanocalorimetry and nanofocus X-ray diffraction analyses. The advantage of using nanofocus 2D WAXS over conventional WAXS has been demonstrated by detecting the change from homogeneous to heterogeneous crystal nucleation regimes. Our study of real-time isothermal melt crystallization of iPP reported here provides novel insights that may be useful for the engineering and processing of this industrially important polymer.

## Data Availability

The data presented in this study are available on request from the corresponding authors.

## Supplementary Materials

The following are available online at https://www.mdpi.com/article/10.3390/nano11102652/s1, Figure S1: Two-dimensional WAXS image of the iPP fibre material used; Figure S2: Decomposed 1D WAXS curve for CR_M1 morphology; Figure S3: Heating endotherms of iPP mesophase formation at rates between 40 and 2000 K s^−1^.
