# Supplementary material for: Assessing Fast Structure Formation Processes in Isotactic Polypropylene with a Combination of Nanofocus X-ray Diffraction and In Situ Nanocalorimetry
Source: Nanomaterials (Basel). 2021 Oct 9;11(10):2652. doi: 10.3390/nano11102652 (PMC8541291; doi:10.3390/nano11102652)
Supplement: Supplementary file 1 [file nanomaterials-11-02652-s001.zip › nanomaterials-1374722-supplementary.pdf]

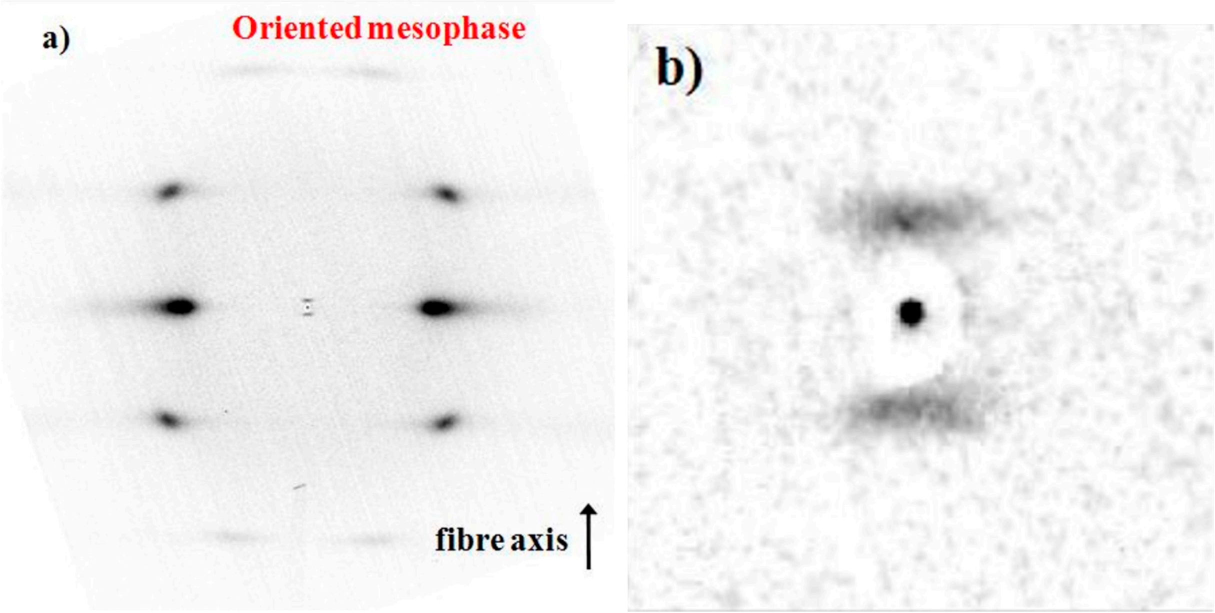

Figure S1. 2D WAXS of iPP fibre. (**a**) Highly oriented mesophase; (**b**) Close view of the small angle region with SAXS signal.

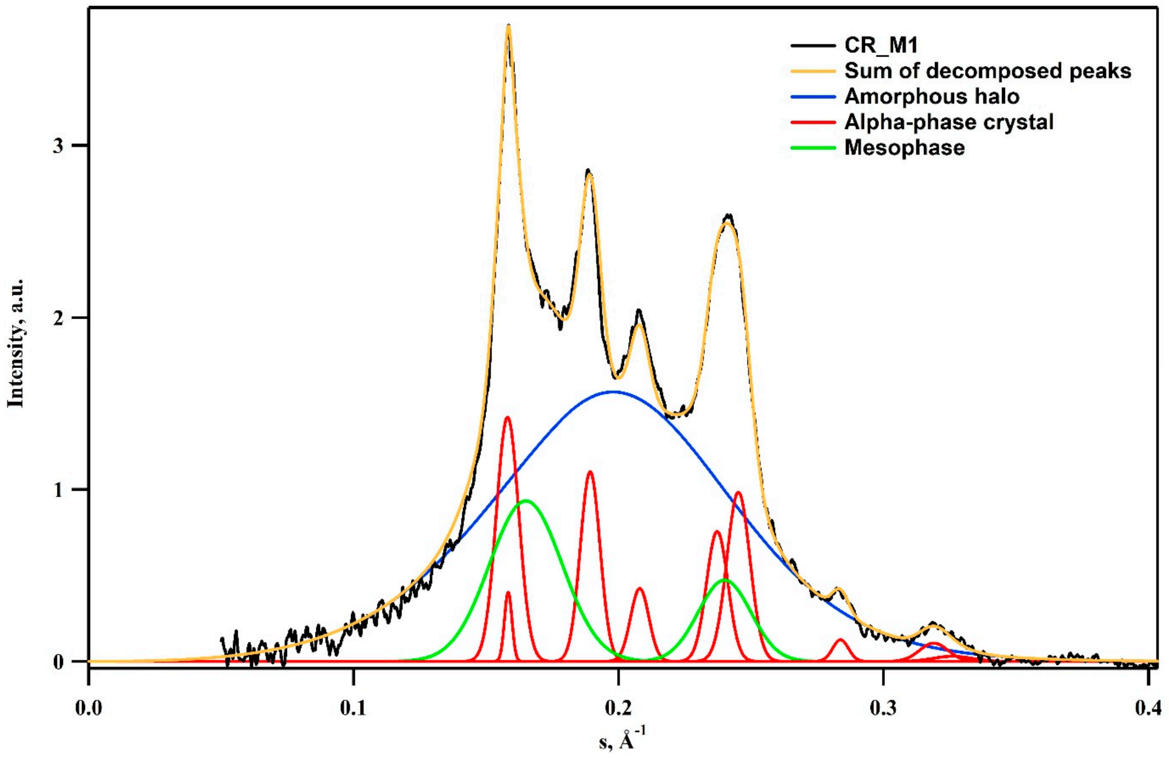

Figure S2. Example of decomposition of the 1D WAXS curve for CR_M1 state showing the mesomorphic (green), crystalline (red) and amorphous (blue) components, the summed intensity of all the components (yellow) as well as the experimental data (black).

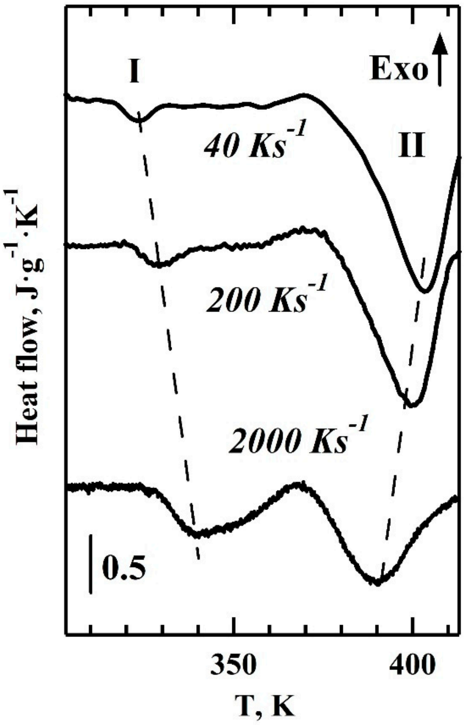

Figure S3. Heating endotherms of iPP mesophase at different rates (T_{max}=413 K). All the endotherms are normalized by the sample mass and the heating rates.
